# Observations and Personal Experience on the Improved Technique of Nitrous-Oxide-Oxygen and the Ether Vapor Anaesthesia

**Published:** 1912-03

**Authors:** Thomas J. Collier

**Affiliations:** Atlanta, Ga.


### OBSERVATIONS AND PERSONAL EXPERIENCE ON THE IMPROVED TECHNIQUE OF NITROUS-OXIDE-OXYGEN AND THE ETHER VAPOR ANAESTHESIA.

By Thomas J. Collier, M. D., Atlanta, Ga.

I wish to present some features of the new and improved technique of the vapor and nitrous-oxide and oxygen anaesthetics, which are not, probably, generally well known.

The vapor apparatus I use was devised by Dr. Jas. T. Gwathmey, of New York, and bears his name—The Gwathmey Vapor Inhaler. It is composed of three bottles, two of which are graduated, one in ½ ounce up to 4 ounces for ether; the other in ½ dram up to 10 drams for chloroform. The top is composed of one stop-cock, or index plate, so arranged that a known per cent. of air and ether, ether and chloroform, chloroform and air, or ether alone, chloroform alone, or air alone, may be given by compressing a bulb, or foot pump, with each inhalation. The air passes down a tube which reaches the bottom of the bottle, the tube being flared out at the bottom and perforated with a number of very fine holes. As the air passes out through these holes, the ether is vaporized, rises and passes out through an opening at top of bottle which leads to a tube in the bottle containing warm water; this tube is also flared at the bottom, with a number of openings through which the air mixed with ether escapes into the warmed water; this rises to the top of the bottle, with an opening and a tube on the outside of plate leading to face mask, which is an ordinary Esmark mask with a tubular base perforated with a few holes on the inner side, to allow exit of warmed vapor. The chloroform goes through the same process, except the tube here has not more than one-sixth the number of perforations as the ether tube. There is also a tube to be used in place of the face mask for operations about the nose, mouth and throat, such as cleft palate work, adenoids, tonsils, etc. Also a mouth gag for the same purposes.

I have observed that with the use of this apparatus, one can produce a very light, at the same time regular, narcosis, owing to the fact that the patient gets the warmed vapor on each inhalation as the bulb or pump is compressed just at the beginning of inspiration. Consequently, the patient gets practically all the vapor and not half of it blown off over the room on exhalation. It takes very much less anaesthetic than any other method I have used, the average being four ounces of ether to the hour, while some take this amount with a dram of chloroform. By a glance at your index, you can tell just what per cent. of proportion of ether and choloform your patient is getting. The warmed vapor is not so irritating to the mucous membranes, consequently, less danger of ether pneumonia and bronchitis following. Neither is it so nauseating as when given by the open drop or closed methods; not so likely to have kidney or liver complications, because the system is not so thoroughly saturated with the drugs. As I have said before, this produces very light narcosis. I have had patients begin to show signs of reacting in three or four minutes after removing the mask. As Dr. Gwathmey says, "You can keep your patients almost within calling distance all the time." Of course, one can produce most profound anaesthesia by increasing the force of compression on the bulb, and giving full ether, or ether and chloroform in large proportions.

But more particularly do I wish to call the Society's attention to the nitrous-oxide and oxygen apparatus, and the administration of these gases as a general anaesthetic. The apparatus that I have was devised by Dr. R. P. Peairs, of Milwaukee, Wis. I found, after using it on several patients, some disadvantages in not have an attachment for using ether if desired. My experience has been in certain cases, especialy in alcoholics and athletes, that it is hard or almost impossible to get complete muscular relaxation without the assistance of a little ether or chloroform. To this apparatus I have added an ether attachment. I can give any amount of ether, from a very slow drop to a stream, to overcome the muscular rigidity; or a small quantity mixed with the gases to reduce their expense. When ether is added we do not have a true nitrous-oxide and oxygen anaesthesia. I have found even the addition of small quantities of ether increases the tendency to nausea and vomiting. The ether attachment is the Gotch Ether Mixing Chamber, of the Gotch Nitrous-Oxide-Oxygen-Ether Apparatus, of Baltimore, with some changes so as to fit my apparatus. I have also added the Teter face mask, which has a positive pressure valve that can be so adjusted as to get any amount of rebreathing, from no rebreathing to full rebreathing, if desired. To my mind, partial rebreathing is quite an advantage, as it diminishes waste and helps to warm the gases in the bags before next inhalation. The warmed gases are more readily taken up by the blood than cold, consequently not so great an amount of the gas is required to produce and maintain narcosis. The warmed gases are less chilling to the mucous membrane of the respiratory tract than the unwarmed gases, and as we do not want to chill our patient, especially in conditions where we usually use nitrous-oxide and oxygen, I think the warming chamber in connection with the apparatus quite an advantage.

I do not think this an ideal anaesthetic as a routine, especially in the hands of every surgeon, even with an expert as an anaesthetist; but is a splendid anaesthetic for the very delicate and painstaking surgeon, as I have noticed the more tenderly the tissues are handled, especially those of the abdominal cavity, the easier and smoother the anaesthesia, together with a marked reduction in the amount of shock, which is the most important of all. It has been clearly demonstrated by Dr. George W. Crile, of Cleveland, Ohio, that rough handling and manipulation arouse the brain cells, and to quote R. J. Morris, "It seems to me that the object lesson of the result of conserving the patient's natural resistance, has opened the vista of a new epoch in surgery. Our faces are now turned toward Metschnikoff and Wright with their descriptions of phagocytes and opsonins, and of the natural protective force of the patient. We are to conserve the natural resistance of the patient and turn him over to his phagocytes and antibodies as helpful as we can. This is the new principle—turning the tide of battle only, and leaving the patient with his phagocytes as nearly intact as possible. It is quite as important for the patient to retain his naural resistance after operation, as it is for the surgeon to add artificial means for securing asepsis during operation. "The first stage of surgery was heroic, the second was anatomic, the third was pathologic and we are now about to enter upon the fourth, or physiologic stage of surgery. Immunity is to be the watchword of the day. Behold the dawn of the fourth era of surgery lighting up the horizon!" (Extract from the Fourth Era of Surgery.) In all septic and toxic cases, where the patient needs all his phagocytes, I know of no other anaesthesia that equals or approaches that of nitrous-oxide and oxygen. My limited experience has shown such a marked difference in the immediate and after effects on the patient, that to my mind, this is the anaesethic of choice in such cases. The patient usually awakes in a few minutes after removal of the mask; does not have the depression usually shown after other forms of anaesthetics, nor the nausea which is a source of great and positive danger to the patient. They are usually able to carry on an intelligent conversation before leaving the operating room, and begin to improve immediately with toxines. The ages of the patients I have used this anaesthetic on range from nine months to 95 years, and for almost every condition met with in surgery.

The administration of this agent requires more skill and practice on the part of the anaesthetist than other anaesthetics to give an even, smooth anaesthesia, and to keep relaxation, at the same time the proper percentage of oxygen to keep the patint in a pink glow. I find that the average person takes about five to six per cent. of oxygen. The expense per hour averages six to seven dollars—somewhat higher than with ordinary anaesthetics, but this increase in cost is more than compensated by the reduction of mortality in septic and asthnic conditions generally, by the comfort of the patient, and by the reserve and conservation of all his vital forces.

Peters Bldg.

---

*Discussion of Dr. Collier's Paper*:—

Dr. W. A. Selman said he had used a modified Brophy inhaler and sent a mixture of ether and chloroform with a rubber tube through the nose for 4 hours and kept the patient within "calling distance" all the time. The degree of perspiration, the pulse and color will give sufficient guides to this nice balancing in long continued anaesthesias. This method is especially valuable in hair-lip operations.

As to gas-oxygen, we have used it a long time as preliminary to ether and for short operations. It requires close watching but the gas can be mixed with air successfully in a Bennett inhaler. He approved of Collier's modified apparatus.

Dr. J. B. Baird, Jr., asked if there were increase of arterial pressure in the rebreathing method.

Dr. H. M. Lokey said the usual open method is not satisfactory in operations on the nose or throat because one has to stop so often to renew anaesthesia. In tonsillectomy the Gwathmey method has been practical and of great advantage. He has had one case in which dissection was necessarily prolonged many minutes and the patient kept asleep by this method. He recovered almost immediately after the vapor was withdrawn and there was no shock.

Dr. F. W. McRae said that anaesthesia was fully as important as any part of surgery, if not the most important. We are getting away from ether and chloroform in toxic cases. He now operates on patients under nitrous-oxide-oxygen anaesthesia that otherwise would have to die, because he can with this anaesthesia so conserve the phagocytes as to prevent toxic death which would ensue if ether or chloroform were used. Crile has done thousands of such cases successfully and McRae has done many himself. The mixture should be carefully made and kept warm. It is a great comfort to him to have an anaesthetist who thoroughly understands his business.

Dr. E. G. Ballenger commended the method, especially in prostatectomy in the aged. One old man in wretched condition whose bronchus was much dilated with a profuse expectoration stood this operation nicely.

Dr. Collier closed, saying that the blood pressure is raised and the method is contraindicated in atheroma.

Local anaesthesia of parts may be used in advance of the vapor and by blocking off the sensations sent to the brain in this manner, shock is lessened.

In his 70 cases, the average time has been 27 minutes; the longest was 1 hour and 50 minutes.

